# Tailoring a facile electronic and ionic pathway to boost the storage performance of Fe_3_O_4_ nanowires as negative electrode for supercapacitor application

**DOI:** 10.1038/s41598-024-66480-5

**Published:** 2024-07-22

**Authors:** Ahmed M. Abdelrahim, Muhammad G. Abd El-Moghny, Hosam H. Abdelhady, Hager S. Wali, Mariam M. Gamil, Samanta R. Fahmy, Toka M. Abdel-Hamid, Gehad K. Mohammed, Yasmeen A. Ahmed, Mohamed S. El-Deab

**Affiliations:** Department of Chemistry, Faculty of Science, Cairo University, Cairo, Egypt

**Keywords:** Graphite felt, Surface fluctuations, Fe_3_O_4_, Negative electrode, Supercapacitor, Electrochemistry, Energy

## Abstract

Today, high-energy applications are devoted to boosting the storage performance of asymmetric supercapacitors. Importantly, boosting the storage performance of the negative electrodes is a crucial topic. Fe_3_O_4_-based active materials display a promising theoretical storage performance as a negative electrode. Thus, to get a high storage performance of Fe_3_O_4_, it must be tailored to have a higher ionic and electronic conductivity and outstanding stability. Functionalized graphite felt (GF) is an excellent candidate for tailoring Fe_3_O_4_ with a facile ionic and electronic pathway. However, the steps of the functionalization of GF are complex and time-consuming as well as the energy loss during this step. Thus, the in-situ functionalization of the GF surface throughout the synthesis of Fe_3_O_4_ active materials is proposed herein. Fe_3_O_4_ is electrodeposited at the in-situ functionalized GF surface with the crystalline nanowires-like structure as revealed from the various analyses; SEM, TEM, Mapping EDX, XPS, XRD, wettability test, and Raman analysis. Advantageously, the synthetic approach introduces full homogeneous and uniform coverage of the large surface area of the GF. Thus, Fe_3_O_4_ nanowires with high ionic and electronic conductivity are characterized by a higher storage performance. Interestingly, Fe_3_O_4_/GF possesses a high specific capacity of 1418 mC cm^−2^ at a potential scan rate of 10 mV s^−1^ and this value retained to 54% at a potential scan rate of 50 mV s^−1^ at an extended potential window of 1.45 V. Remarkably, the diffusion-controlled reaction is the main contributor of the storage of Fe_3_O_4_/GF electrode as revealed by the mechanistic studies.

## Introduction

Nowadays, supercapacitors (SCs) have boomed in their usage as storage devices in many high-energy applications such as smart devices and electric transportation. Also, the increasing call for energy storage devices becomes a need to reserve the surplus energy resulting from various renewable energy sources to be used on demand^1–4^. Thus, for thriving SCs, the researchers directed their efforts to engineering materials characterized by high specific capacity (*C*_*s*_), wide potential window, and outstanding stability that leads to higher storage performance in terms of energy and power densities^5–7^.

According to the materials’ type as positive and negative electrodes, the SCs can be categorized into two key categories, symmetric and asymmetric devices. The asymmetric device has the advantage of possessing higher output energy and power density due to having an extended potential window via employing two dissimilar materials that operate in different voltage regions^8–10^. Today, there are many characteristics of materials used in the positive potential region. In contrast, the negative potential region is mainly limited to carbon-based substances. Nevertheless, the resulting energy from the carbon-based materials is low due to it is mainly based on the accumulation of the charge on its surface. Thus, the researchers started to investigate other materials for SCs applications that are functioning in the negative voltage region with high energy. Engineering electrode materials functioning in the negative voltage region with a faradaic reaction is a promising solution for this problem due to their high capacities^11,12^.

Fe-based materials show a superior performance as negative electrodes due to their operating along wide potential windows via transition between their various oxidation states resulting in high capacities. Additionally, securing low-cost, safe use, environmentally friendly, and natural abundance materials strengthen their usage in SC applications. Fe can exist in various forms such as Fe_3_O_4_, Fe_2_O_3_, and FeOOH^11–14^. Among them, Fe_3_O_4_ has a great advantage over the others because it has high electronic mobility due to the delocalization of the electrons between Fe^2+^ and Fe^3+^ oxidation state^15^. But Fe_3_O_4_'s electrical conductivity is still not high enough, thus more modifications are needed to robust its electronic conductivity to be used as efficient negative electrode materials^16,17^. Accordingly, many attempts are explored to speed up the transfer of ions and electrons within Fe_3_O_4_ materials such as (i) tailoring-design of the active substances at the surface of porous and conductive supports or in the presence of conductive additives, (ii) preparation of Fe_3_O_4_ active materials in nanostructures such as nanosheets, nanowires, nanorods, and nanosphere, and (iii) preparation of the active materials without any binder.

Xuezhen Zhu et al.^18^ synthesized Fe_3_O_4_ at the nitrogen-doped carbon support and the prepared electrode displayed a pseudocapacitive performance with a high specific capacitance of 206 F g^−1^. Also, Zafer Çıplak^19^ proposed a core–shell structure by surrounding the Ag nanoparticles, as a conductive additive, with Fe_3_O_4_ redox active materials having pseudocapacitive behavior. The synthesized electrode showed a high specific capacitance of 212.2 F g^−1^. Jiehao Guan^20^ effectively tailored Fe_3_O_4_ in nanosheets-like morphology at the surface of reduced graphene oxide with a pseudocapacitive property and specific capacitance of 442 F g^−1^. Moreover, Haiqiang Luo^21^ fabricated FeOOH/Fe_3_O_4_ hydrothermally at the surface of the stainless-steel support as a binder-free method and the electrode exhibited a battery-like behavior with a high *C*_*s*_ of 396 mA h g^−1^. Importantly from the mentioned examples, the origin of the storage performance of Fe_3_O_4_ redox active materials may behave as pseudocapacitive or battery-like electrodes according to the speed of the redox reaction with the electrolyte’s ions. Additionally, the terminology of the specific capacitance is used for electrical double-layer capacitors and pseudocapacitive behavior in a unit of F g^−1^. Specific capacity in a unit of C g^−1^ or mA h g^−1^ is used for the battery-like behavior (as the material used herein)^22–25^. Thus, precise terminology must be used for the proposed materials. Additionally, the differentiation between pseudocapacitive behavior and battery behavior can be done using cyclic voltammetry (CV) and galvanostatic charge–discharge (GCD) techniques because each behavior has its characteristic shape. The pseudocapacitive behavior is characterized by an approximately rectangular CV shape, and an approximately linear GCD curve with time^26–28^ (relatively like electrical double-layer capacitor behavior, e.g. graphene^29^). The battery behavior is signified by characteristic peaks within a small voltage difference in CV and plateau in GCD^26,27^.

As mentioned above, using the conductive porous support will accelerate both the electron and ion transfer. Advantageously, the porous and conductive carbon-based support outstands as a promising support owing to its low-cost, being environmentally friendly, safe, and having good physical and chemical properties^30,31^. Among the various carbon-based supports, graphite felt (GF) has the potential strength to boost the capacitive performance of the active substances owing to their 3-D porous structure that is characterized by high electrical conductivity and large surface area^32–34^. However, the ionic accessibility inside the 3-D matrix of GF is hindered by the high degree of hydrophobicity. But this problem is overcome by the insertion of polar functional groups such as OH^−^, C=O, and COO^−^. Synthesizing the active substances at the functionalized GF surface assists the easy contact between active materials and electrolyte ions by accelerating the ions' mobility and providing a homogenous structure. Furthermore, the cyclic performance of the active materials throughout the charging and discharging over the high cyclic number is greatly enhanced due to the functionalized surface strength and the contact with the active materials^5,6^.

Accordingly, this study aims to propose a preparation method with multiple purposes in one step. The first purpose is the preparation of Fe_3_O_4_ in the nanostructure morphology to increase the ionic and electronic conductivity. Secondly, structure flaws are introduced via inserting the functional groups on the GF surface to provide homogenous and uniform distribution and accelerate the contact of the active materials with electrolyte ions. The third one is to make the interface joint between the nanostructure Fe_3_O_4_ and the functionalized GF surface without any binder. Finally, covering a huge surface area of the GF support and obtaining high areal capacity are achieved. Nanostructured Fe_3_O_4_ is electrochemically prepared on the functionalized GF surface via sweeping the GF support from the oxidative to the reductive potential region in the solution containing various concentrations of FeCl_3._ The nanowire structure of Fe_3_O_4_ and GF surface functionalization are approved by various characterization analyses. Advantageously, the Fe_3_O_4_/GF electrode possesses a distinctive storage performance over a wide potential window. Moreover, the mechanistic study discloses that the storage mechanism of the Fe_3_O_4_/GF electrode is the combination of the fast surface redox reaction and the diffusion-limited reaction. However, the predominated mechanism is the limited diffusion reaction, and this is clear from the shape of the cyclic voltammogram (CV) and the galvanostatic charge–discharge (GCD) curve. Fe_3_O_4_/GF electrode displayed a *C*_*s*_ of 1418 mC cm^−2^ at a potential scan rate of 10 mV s^−1^ and this value retained at 54% at a potential scan rate of 50 mV s^−1^ demonstrating an outstanding capacitive performance. Interestingly, the operational voltage window of the synthesized electrode is one of the highest voltage windows for Fe-based materials.

## Experimental

### Materials

All the compounds used were of analytical grade and obtained from Merck, and they were all used exactly as supplied without any additional purification. Second distilled water was used to prepare the solutions.

### Synthesis of Fe_3_O_4_-based electrodes

All electrodes were prepared by electrodeposition using a three-electrode configuration; working electrode (GF with dimensions 4 mm × 4.6 mm × 2 mm), counter electrode (graphite rod), and reference electrode (saturated calomel electrode (SCE)).

1. Using the same deposition bath (100 mM FeCl_3_), three electrodes were prepared using the linear sweep voltammetry (LSV) setup at potential windows: (2 to − 2 V), (2 to − 3 V), and (2 to − 4 V).
2. Using the same deposition potential window (2 to − 4 V), two other electrodes were prepared via the LSV technique using a deposition bath with various concentrations (150 mM, and 200 mM).
3. Using cyclic voltammetry (CV) setup, all the prepared electrodes are activated in 1 M KOH solution via cycling the potential from 0 to − 1.45 V for 25 cycles at a potential sweep rate of 10 mV s^−1^.

Note that: the best-optimized electrode (Fe_3_O_4_/GF) is the electrode that is prepared in a solution containing 200 mM FeCl_3_ at a potential window (2 to − 4 V)_._

### Physical characterization

A field emission scanning electron microscope (SEM) (JEOL, Japan JSM IT-100) in conjunction with an energy dispersive X-ray spectrometer (EDX) device was conducted to probe the surface morphology and chemical composition of the modified electrodes. Transmission electron microscopy (TEM) and high-resolution electron microscopy (HR-TEM) (JEM-HR-JEOL-JEM 2100, Japan) were conducted for the investigation of the material’s morphology and the material’s crystal structure. X-ray photoelectron spectroscopy (XPS) was also probed to disclose the electrodes’ surface composition (using monochromatic X-ray Al K-alpha radiation, Thermo Fisher Scientific). To further analyze the crystalline structure, X-ray diffraction (XRD) with Cu K_*α*_ radiation, STOE STADI, was used. Furthermore, the Attention Biolin Scientific (Version 2.7) wettability test was probed to explore the contact angle of the prepared materials, i.e., surface hydrophilicity. Moreover, Raman spectra captured by Lab RAM HR Evolution were employed to monitor the presence of the redox-active components and the level of surface modification of the GF support.

### Electrochemical measurements

The storage performance of the synthesized active materials was determined from the relevant electrochemical measurements operated using a Biologic Potentiostat (VSP-300) at room temperature (25 °C ± 1) and electrolyte solution containing 1 M KOH. electrochemical tests: CV, galvanostatic charge–discharge (GCD, and electrochemical impedance spectroscopy (EIS) (at open circuit potential from 100 kHz to 10 mHz) measurements were carried out in three-electrode cell (Fe_3_O_4_/GF is the working electrode, graphite rod is the counter electrode, and SCE is the reference electrode) to evaluate the storage performance of the as-synthesized materials.

The *C*_*s*_ calculated by Eq. 1 and 2 from the integrated area under the CV and GCD curves, respectively^33,35,36^:

1 $$C_{s} \left( {{\text{C cm}}^{ - 2} } \right) = \frac{{\smallint Idt{ }\left( C \right)}}{{S{ }(cm^{2} )}}{ } = { }\frac{{\smallint IdV{ }}}{{v{ }}}{ }\left( C \right)\frac{1}{{S\left( {cm^{2} } \right)}}$$

2 $${C}_{s}=\frac{\int I\Delta t (C)}{S ({cm}^{2})}$$

where *C*_*s*_ is the specific capacity, *∫IdV/v* or *∫Idt* is the total charge, *S* is the electrode geometric surface area (GF (6 faces) has S = 0.712 cm^2^), *∫I*Δ*t* is the total charge, and Δ*t* is the discharge time.

## Results and discussion

### Optimization of GF surface alternation and Fe_3_O_4_ deposition

**Figure 1:**
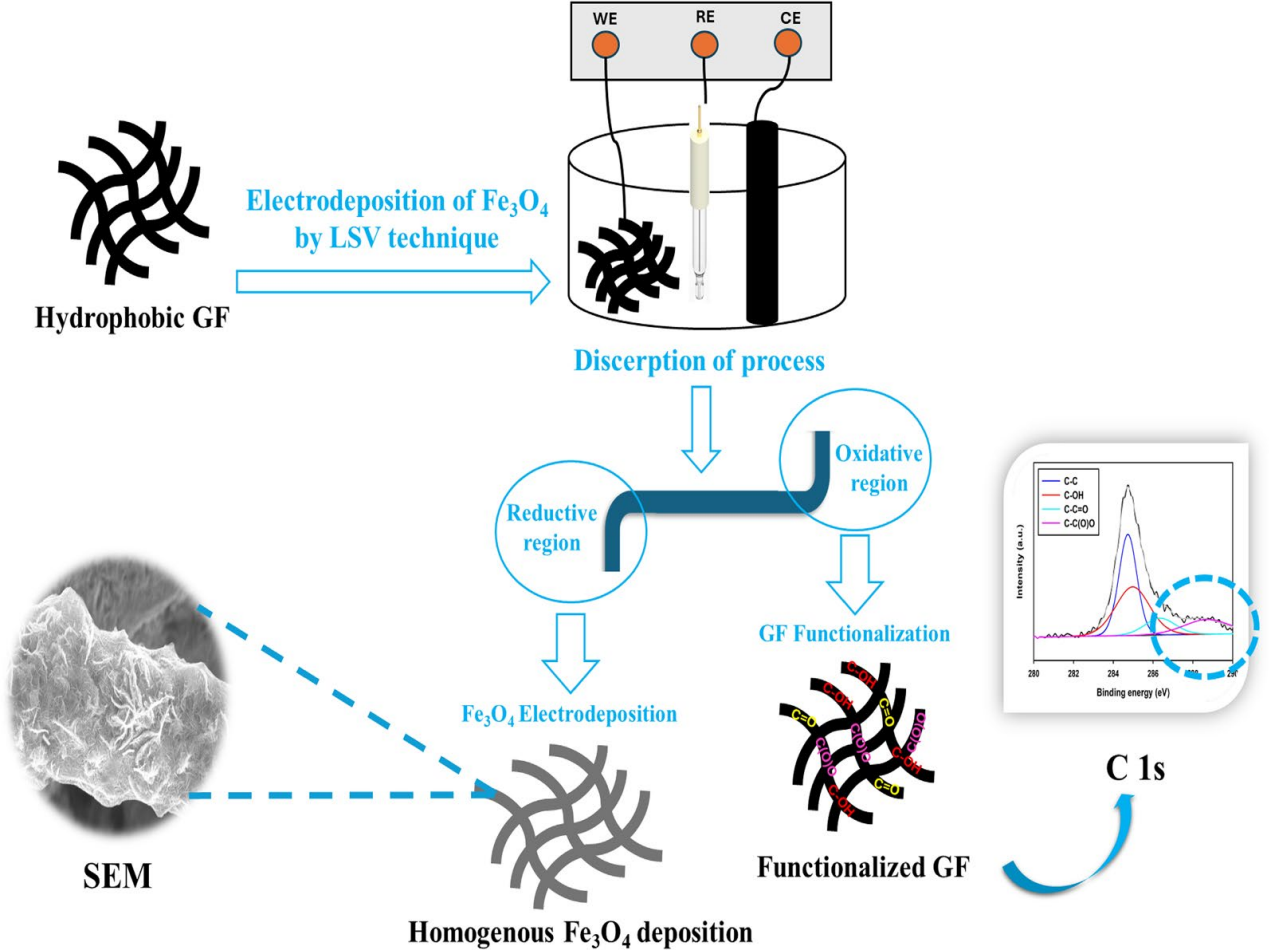
Graphic illustration of the synthesis of Fe_3_O_4_/GF electrode.

Herein, the multifunction one-step method is proposed to synthesize Fe_3_O_4_ in the crystalline nanostructure over the in-situ functionalized GF surface with high areal capacity. The GF functionalization and Fe_3_O_4_ electrodeposition are done by sweeping the potential from the oxidative potentials (positive potentials) followed by the reductive potentials (negative potentials) via the LSV technique, respectively, in the deposition bath containing FeCl_3_. While the role of the oxidative potentials is to functionalize the GF surface, the role of the reductive potentials is to deposit the Fe_3_O_4_. Functionalization of hydrophobic GF surface as seen from Fig. 1 occurred at the oxidative potentials with the aid of the formed oxygen, H_2_O, and ClO_3_^−^ species that are in-situ formed at high positive potential^32,37–39^. The inserted oxygenated functional groups at the GF surface were probed by various surface characterization tools, particularly XPS studies (*c.f.* Figure 3). Additionally, the in-situ functionalized GF assisted in the homogenous uniform deposition of Fe_3_O_4_ redox active material around almost all GF fibers (*c.f.* Figure 5). The formation of the Fe_3_O_4_ is started in the reductive region by the reduction of Fe^3+^ to Fe^2+^ followed by the formation of Fe_3_O_4_ assisted by the hydrogen evolution reaction at the electrode surface according to the following Eqs. (3–5)^39,40^:

3 $${Fe}^{3+}+{e}^{-}\to {Fe}^{2+}$$

4 $$2{H}_{2}O+2{e}^{-}\to {H}_{2}+2{OH}^{-}$$

5 $${Fe}^{2+}+2{Fe}^{3+}+8{OH}^{-}\to {Fe}_{3}{O}_{4}+4{H}_{2}O$$

**Figure 2:**
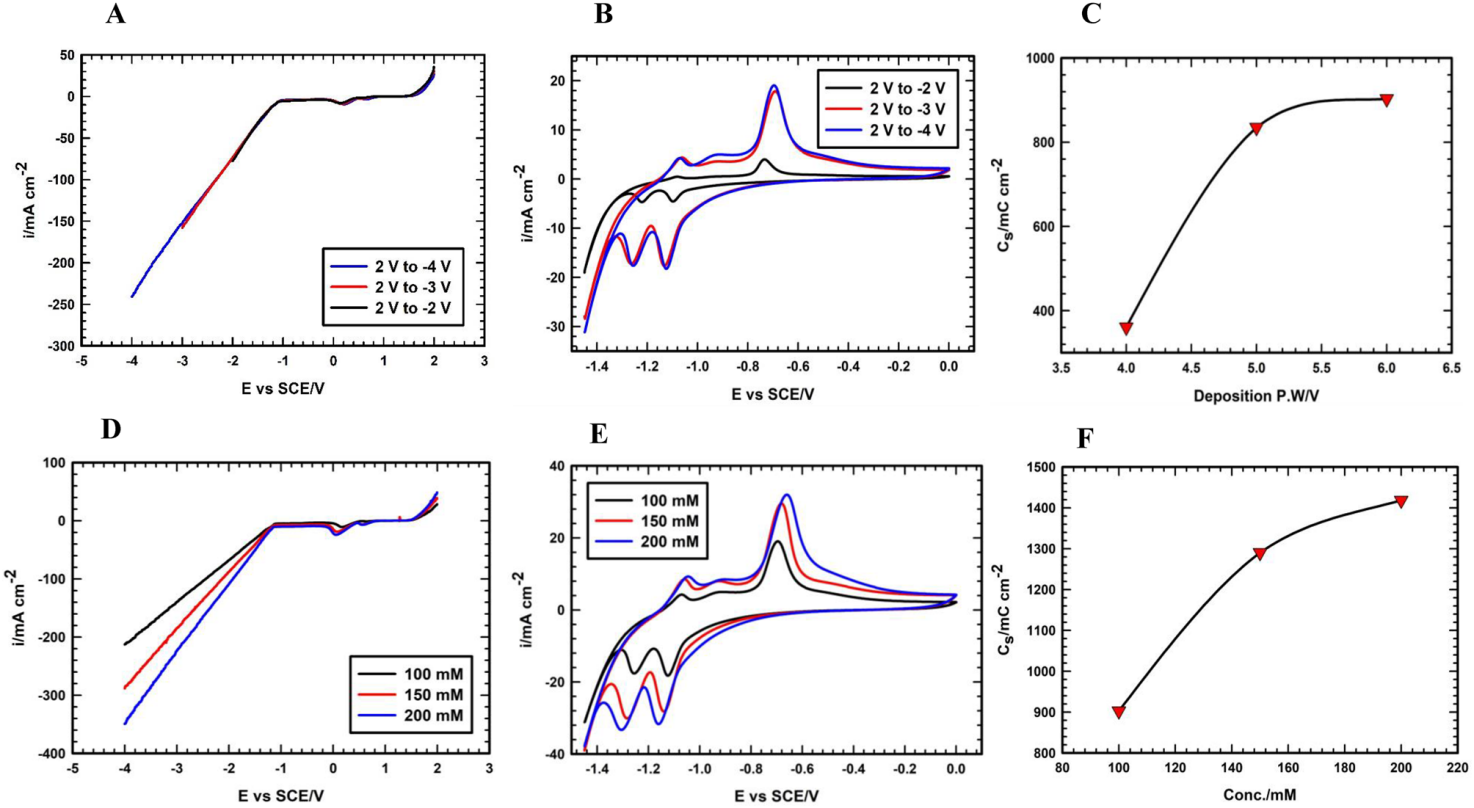
(**A**) LSVs, (**B**) CVs, and (**C**) variation of C_s_ with varying deposition potential window curves of the electrodes prepared at potential windows of (2 to − 2 V), (2 to − 3 V), and (2 to − 4 V). (**D**) LSVs, (**E**) CVs, and (**F**) variations of C_s_ with varying concentrations of deposition bath curves of the electrodes prepared using various ferric chloride concentrations (100, 150, and 200 mM). Note that: all CV measurements were performed in 1 M KOH solution at a potential scan rate of 10 mV s^−1^.

Thus, according to the above-mentioned idea, the conditions were optimized by investigating the width of the potential window together with the electrolyte concentration. Firstly, three electrodes were prepared, using a concentration of the deposition bath equal to 100 mM FeCl_3_, applying various potential windows (2 to − 2 V, 2 to − 3 V, and 2 to − 4 V), see Fig. 2A. Importantly, the amounts of the loaded Fe_3_O_4_ are increased by opening the potential to the higher negative values. As a result, and from Fig. 2B, the electrode that is obtained at the extended potential window (+ 2 to − 4 V) is the best-prepared electrode due to the integrated surface area under the CVs resulting in a higher areal capacity (see Fig. 2C). Further, the effect of the FeCl_3_ concentration at the optimized deposition potential window is investigated using two other concentrations, 150 and 200 mM. Advantageously, as the deposition bath concentration is increased, the level of the functionalization is enhanced, and this is clear from the positive current’s region of all electrodes that is assisting in depositing the active materials in a uniform and homogeneous manner utilizing the maximum available GF surface area (see Fig. 2D). Also, the degree of the deposition of the Fe_3_O_4_ active materials is increased by extending the potentials to the higher reductive potentials, see Fig. 2D. Accordingly, the electrode that is prepared at 200 mM and wide potential window is the best and this is notable from the area under CVs and the calculated areal capacity as shown in Fig. 2E,F. Therefore, the electrode prepared using 200 mM FeCl_3_ solution and applying a potential window from + 2 to − 4V (Fe_3_O_4_/GF) is selected as the best electrode for further physical characterizations and electrochemical measurements.

### Physical characterization

**Figure 3:**
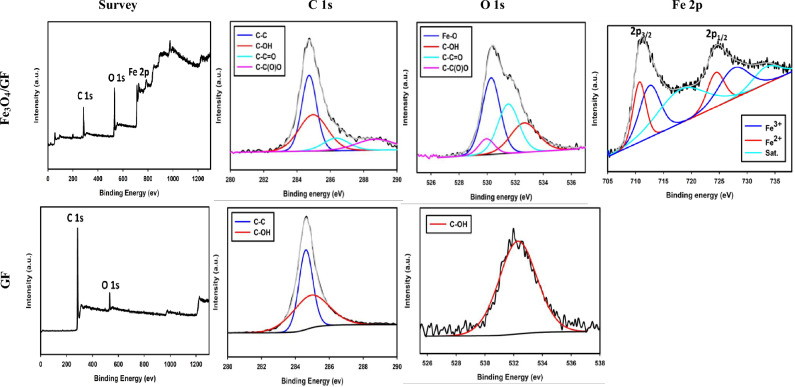
XPS analysis of Fe_3_O_4_/GF and GF electrodes.

XPS analysis is utilized to confirm the existence of Fe in the form of Fe_3_O_4_, and the incorporation of the oxygenated functional groups at the GF surface. From Fig. 3, the incorporation of the Fe element during the synthesis process is clear from the existence of the extra peak around 712 eV in comparison with the survey of pure GF surface. Also, the approval of the Fe_3_O_4_ preparation is verified by the existence of the two oxidation states of iron (Fe^2+^ and Fe^3+^). While the existence of Fe^2+^ is proven by the appearance of two peaks around 711 and 725 eV that are ascribed to the Fe 2*p*_3/2_ and Fe 2*p*_1/2_, respectively, the existence of the Fe^3+^ is proven by the appearance of the peaks around 713 and 728 eV that are assigned to the Fe 2*p*_3/2_ and Fe 2*p*_1/2_, respectively (see Fig. 3)^41–43^. Additionally, from O 1*s* spectra as displayed in Fig. 3, the formation of Fe_3_O_4_ is confirmed by the appearance of a peak assigned to the Fe–O bond^44^. Also, from O 1*s* spectra, one concludes that the successful in-situ GF functionalization is confirmed by displaying more oxygenated functional groups compared to the pristine GF surface. Additionally, the percentage of the O in the Fe_3_O_4_/GF electrode is 4 times that of the GF surface indicating the massive deposition and the in-situ functionalization. Further from C 1*s*, the appearance of the hump at high binding energy around 288 eV indicates the oxidation of the GF surface, and this is clear from the deconvoluted C 1*s* compared to that of the GF surface^32,35,45^.

**Figure 4:**
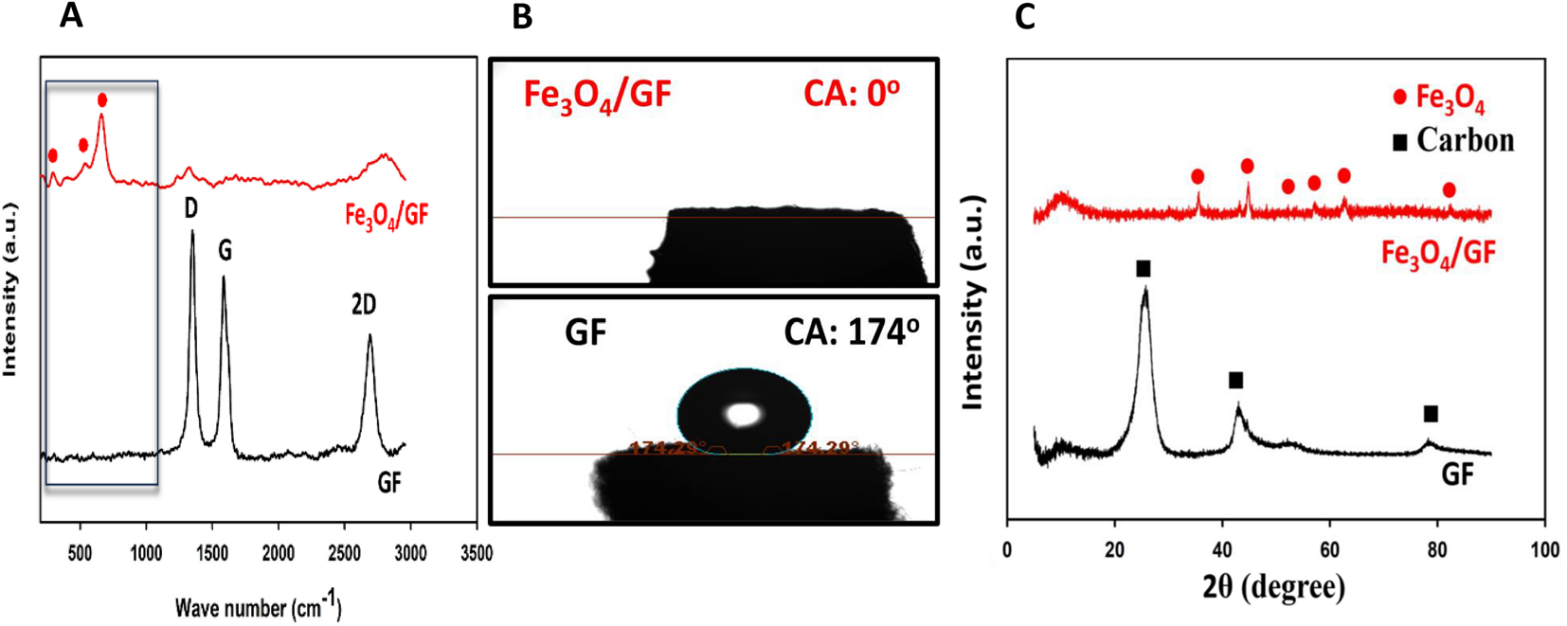
(**A**) Raman spectra, (**B**) contact angle measurements, and (**C**) XRD patterns of GF (black line) and Fe_3_O_4_/GF (red line) electrodes.

Additionally, Raman analysis as a strong characterization tool for differentiation between the iron oxides and oxyhydroxide is done to further confirm the formation of Fe_3_O_4_. The theoretical studies of Raman analysis of the Fe_3_O_4_ materials display several active bands of A_1g_, E_g_, and T_2g_ modes between wavenumbers 200–1000 cm^−1^. From Fig. 4A, the appearance of three bands around 294, 538, and 661 cm^−1^ matches the vibration’s mode of Fe_3_O_4_, which concludes the deposition of Fe_3_O_4_ over the GF surface^46^. Remarkably, the Fe_3_O_4_/GF electrode’s G and D bands vanish, indicating that there is an excessive amount of Fe_3_O_4_ deposited that completely encases the GF fibers (see Fig. 4A).

As a result of the above analysis that confirms the in-situ GF surface fluctuations and heavy deposition of the Fe_3_O_4_ materials, the Fe_3_O_4_/GF electrode shows exceptional hydrophilicity. As displayed in Fig. 4B, the contact angles of GF and Fe_3_O_4_/GF electrodes are 174.29° and 0°, respectively. This improves the capacity performance by smoothing the electrolyte ions' diffusion paths for the reaction with redox-active materials.

XRD analysis is done and boosted the above results. Firstly, from Fig. 4C, the formation of the Fe_3_O_4_ is established by obtaining peaks around 35°, 42°, 52°, 58°, 62°, and 82° assigned to the crystalline structure of Fe_3_O_4_ (JCPDS No. 19-0629)^47,48^. Secondly, as consistent with Raman analysis, the diffraction peaks assigned to the graphitic structure are not present in the Fe_3_O_4_/GF electrode, showing that the active materials have completely covered the fibers of GF with high thickness confirming the massive and homogeneous deposition.

**Figure 5:**
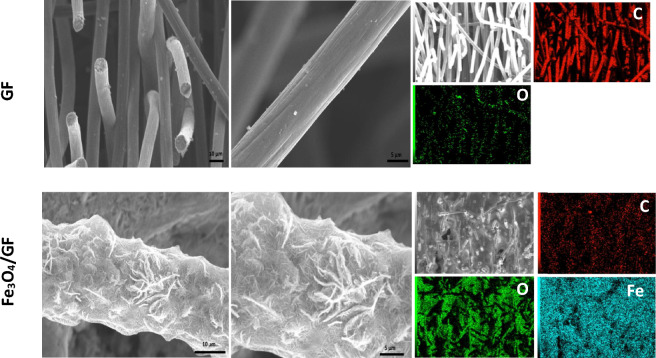
SEM images with various magnifications and the corresponding mapping EDX of GF and Fe_3_O_4_/GF electrodes.

The surface morphology and elements distribution of GF and Fe_3_O_4_/GF electrodes are investigated by SEM and mapping EDX analysis, respectively. From Fig. 5, GF demonstrates a fiber's flawless surface with little white spots that represent carbon dust after manufacture^34,49^. Whereas Fe_3_O_4_/GF, as shown in Fig. 5, exhibits uniform encasement of Fe_3_O_4_ on the in-situ altered GF surface in nanowire shape. Uniform encasement indicates effective surface modification via inserting oxygenated functional groups during the preparation of Fe_3_O_4_/GF which compiles with the findings obtained from XRD and Raman analysis. The complete encasement of GF fibers by active materials and the extraordinary hydrophilicity facilitates the electrolyte ions' pathways toward the active sites predicting a high *C*_*s*_ (*c.f*. Figure 8). Moreover, mapping EDX of Fe_3_O_4_/GF demonstrates the massive deposition of the Fe_3_O_4_ by obtaining a massive amount of Fe element, high O percent, and low C percentage indicating the complete encasement that is consistent with the previous analyses (see Fig. 5).

**Figure 6:**
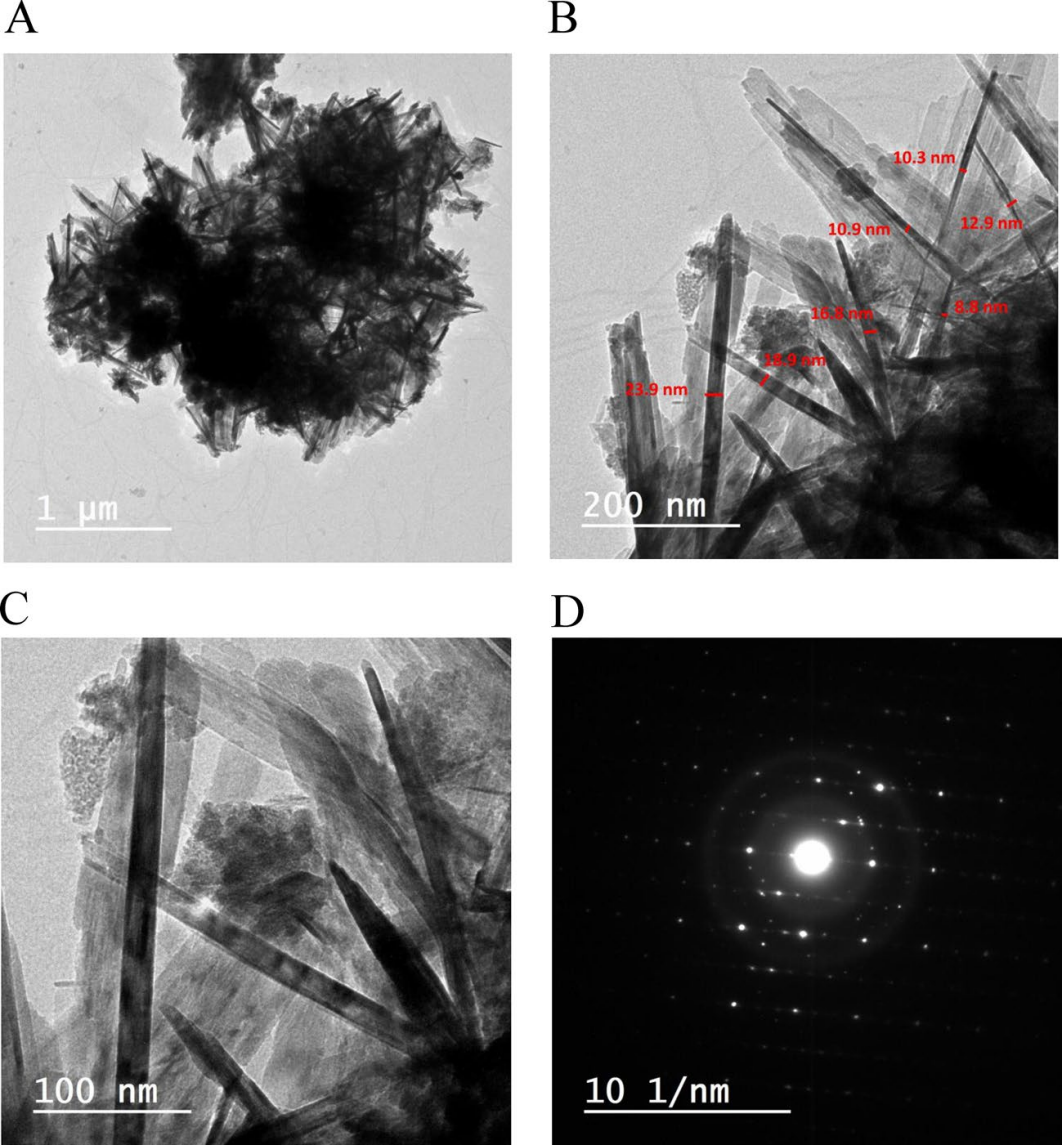
HR-TEM images with various magnifications (**A**–**C**), and (**D**) the corresponding SAED pattern of the Fe_3_O_4_/GF electrode.

TEM and HR-TEM analyses are conducted for Fe_3_O_4_/GF electrode for further confirmation of morphology and crystallinity nature of Fe_3_O_4_ active material. From Fig. 6, TEM images at different magnifications (Fig. 6) display a nanowire morphology with a small diameter (see Fig. 6B) that is consistent with SEM analysis. Furthermore, the Fe_3_O_4_ active particles' selected area electron diffraction (SAED) is shown in Fig. 6D which indicates the polycrystalline nature of Fe_3_O_4_ due to the presence of bright spots surrounding the rings that are consistent with XRD analysis^50,51^.

### Electrochemical storage performance of Fe_3_O_4_/GF electrode

**Figure 7:**
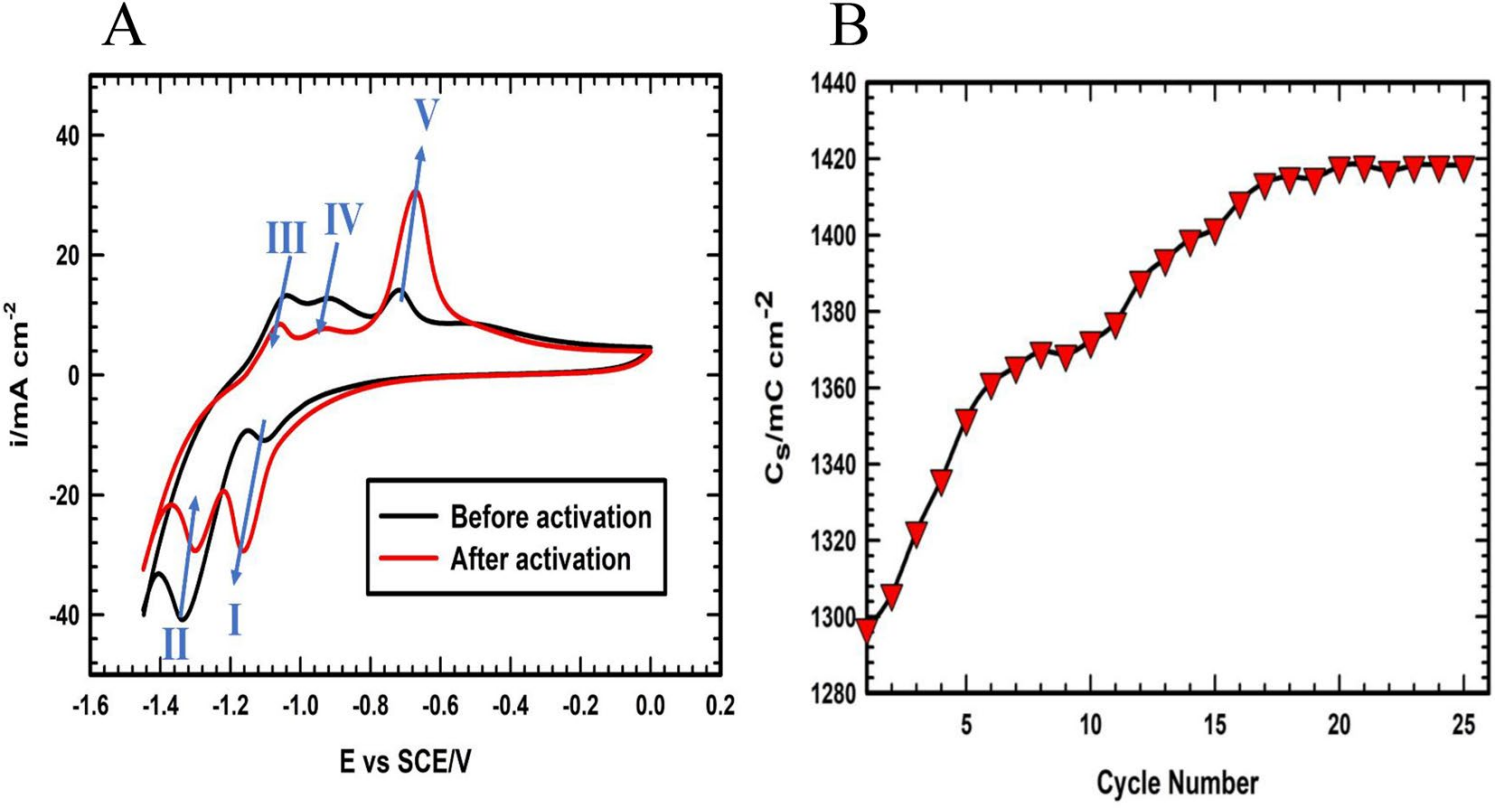
(**A**) CVs of Fe_3_O_4_/GF electrode before (black line) and after (red line) the electrochemical activation. (**B**) The variation of the specific capacity with cycle number during the electrochemical activation of the Fe_3_O_4_/GF electrode.

The peak intensities and the diffusion accessibility of the electrolyte ions to the underneath layers of the active materials vary throughout the first several cycles of operation. Thus, the electrochemical activation must be done to reach a steady state before the evaluation of the electrochemical storage performance of the Fe_3_O_4_ active materials^52,53^. From Fig. 7A, there are characteristic peaks of Fe_3_O_4_ active materials at the cathodic branch (I, and II) and three peaks at the anodic branch (III, IV, and V). During the cathodic direction, the Fe^3+^ is electrochemically reduced to Fe^2+^ (peak I) and then to Fe^0^ (peak II). Moving towards the anodic direction, i.e., toward high potential values, the three characteristics of anodic peaks appeared due to; (a) formation of Fe(OH) by the adsorption (peak III); (b) electrochemical oxidation of the Fe^0^ to the Fe^2+^; and (c) overcharging of the Fe^2+^ to Fe^3+^^52,53^. As shown in Fig. 7A, while the peak intensity of II, III, and IV is decreased by repetitive cycling, the intensity of peaks I and V is increased with cycling. Thus, the activation step is done to reach a steady state and nearly the same behavior, i.e., the same areal capacity (see Fig. 7B).

**Figure 8:**
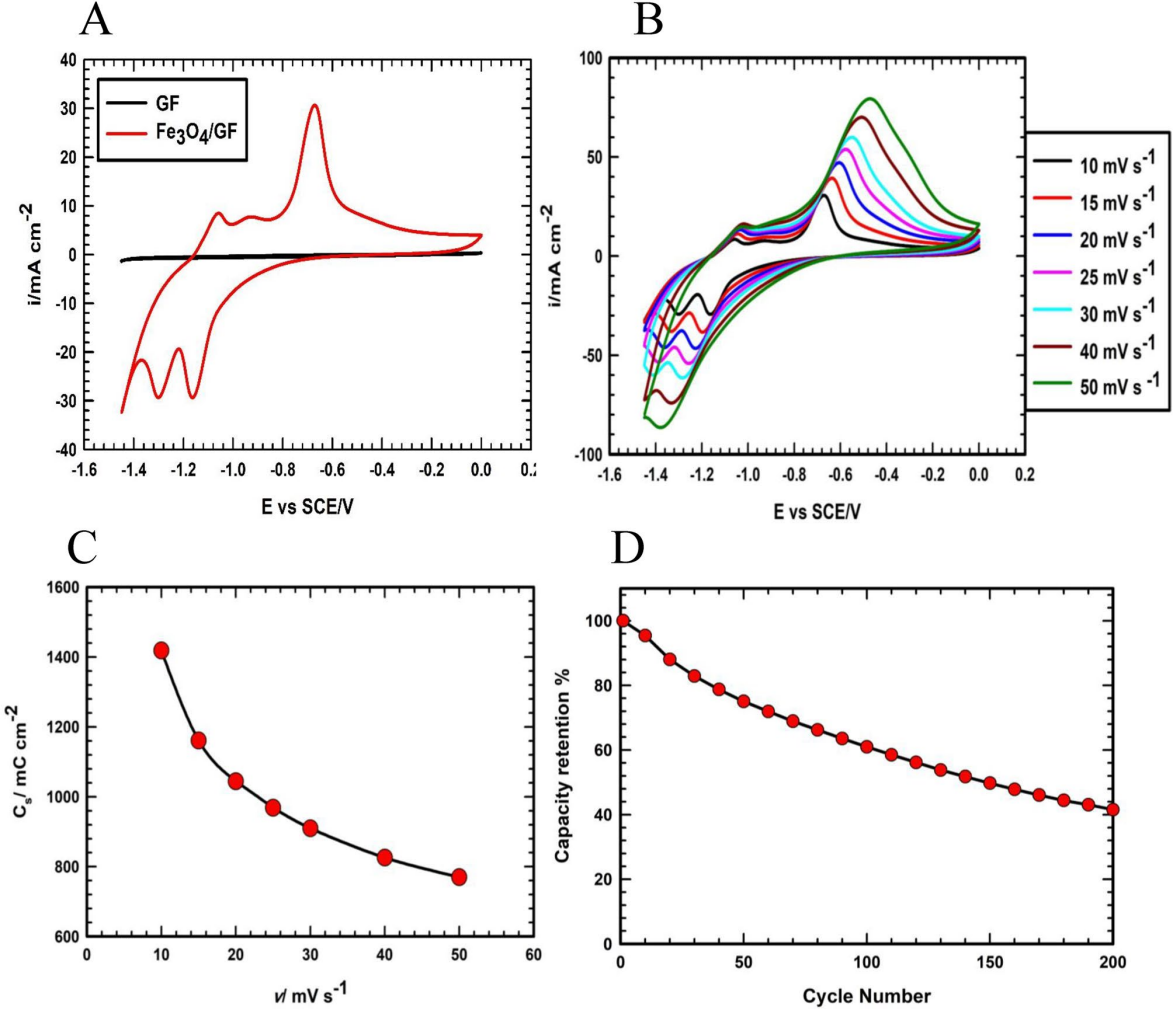
(**A**) CVs of GF (black line) and Fe_3_O_4_/GF (red line) electrodes at a potential scan rate of 10 mV s^−1^. (**B**) CVs of Fe_3_O_4_/GF electrode at various potential scan rates. (**C**) The variation of C_s_ with the variation potential scan rate of the Fe_3_O_4_/GF electrode. (**D**) The capacity retention of Fe_3_O_4_/GF electrode at a potential scan rate of 20 mV s^−1^.

After the electrochemical activation, the electrochemical storage performance of the Fe_3_O_4_/GF electrode is studied utilizing the CV, GCD, and EIS measurements. As seen from Fig. 8A, CVs of Fe_3_O_4_/GF and GF electrodes show that the value of capacity is not significantly influenced by the GF support. However, the electrochemical change of Fe between its oxidation states, as indicated by the peaks in the anodic and cathodic scan, results in a CV of Fe_3_O_4_/GF (red line) with a large surface area indicating a higher capacity, and this reflected the 3-D porous structure of GF support, complete encasement of GF fibers by redox-active Fe_3_O_4_, and the exceptional ionic conductivity of the prepared electrode. The *C*_*s*_ of the Fe_3_O_4_/GF is calculated using Eq. 1 and displays a value of 1418 mC cm^−2^. Advantageously, the *C*_*s*_ value of the Fe_3_O_4_/GF electrode is highly related to other based Fe_3_O_4_ electrodes (see Table 1). Additionally, by using Eq. 1 and normalizing capacity by the loaded mass (9.3 mg cm^−2^) instead of area, the Fe_3_O_4_/GF electrode displays a specific capacity of 153 C g^−1^ which is also higher than other reported values, e.g. Fe_3_O_4_/SDS^54^, CNT/Fe_3_O_4_^55^, Fe_3_O_4_ nanoparticles^56^. Interestingly, one of the significantly effective parameters for the application and performance of SCs is the operational voltage window. Fe_3_O_4_/GF electrode exhibits an extended voltage window up to 1.45 V which is one of the widest potential windows obtained for the storage performance of Fe_3_O_4_ materials in the negative potential region (see Table 1). Additionally, from Fig. 8B, the *C*_*s*_ value of the Fe_3_O_4_/GF electrode is calculated at various potential scan rates. Figure 8C displays the variation of *C*_*s*_ and the obtained values are 1418.3, 1160.7, 1044.2, 968.4, 909.6, 825.6, and 769.6 mC cm^−2^ at various potential scan rates of 10, 15, 20, 25, 30, 40, and 50 mV s^−1^, respectively. The stability test is one of the most crucial factors in determining how well the SC material performs. The stability of Fe_3_O_4_ is shown in Fig. 8D for 200 cycles at a potential sweep rate of 20 mV s^−1^. Fe_3_O_4_/GF exhibits limited stability behavior, which is explained by the mechanical stress that results from the electrolyte ions' intercalation and deintercalation within the active materials during the charge transfer process that occurs throughout the charging and discharging process.

**Table 1:** Comparison of the *C*_*s*_ and the operating potential window of Fe_3_O_4_/GF material with other reported Fe-based materials.

Electrode materials	Electrolyte	C_s_/mC cm^−2^	Potential window/V	References
MXene/Fe_3_O_4_/MXene	1 M Li_2_SO_4_	32.48 @ 0.5 mA cm^−2^	0.7	^57^
Fe_3_O_4_-MoO_2_	1 M Li_2_SO_4_	81.25 @ 2 mV s^−1^	1.25	^58^
Fe_3_O_4_/TiO_2_@C-3	1 M Na_2_SO_3_	380.13 @ 1 mA cm^−2^	1.25	^59^
Fe_3_O_4_-PC	6 M KOH	59.13 @ 20 mV s^−1^	0.55	^60^
CN-Fe_3_O_4_	3 M KOH	399.3@ 1 mA cm^−2^	1	^42^
Fe_2_O_3_/RGO/Fe_3_O_4_	2 M KOH	270@ 20 mA cm^−2^	0.8	^61^
Fe(OH)_3_/Ag/TNTA	1 M KOH	67.736@5 mV s^−1^	0.8	^62^
α-Fe_2_O_3_-Vo	1 M Na_2_SO_4_	862.12@1 mA cm^−2^	1	^63^
α-Fe_2_O_3_/C	1 M Na_2_SO_4_	430.8@1 mA cm^−2^	1	^50^
T-Fe_2_O_3_/PPy	1 M Na_2_SO_4_	305.92@ 0.5 mA cm^−2^	0.8	^64^
Fe_3_O_4_/GF	1 M KOH	1418 @ 5 mV s^−1^ 1300@ 5 mA cm^−2^	1.45 1.2	This work

**Figure 9:**
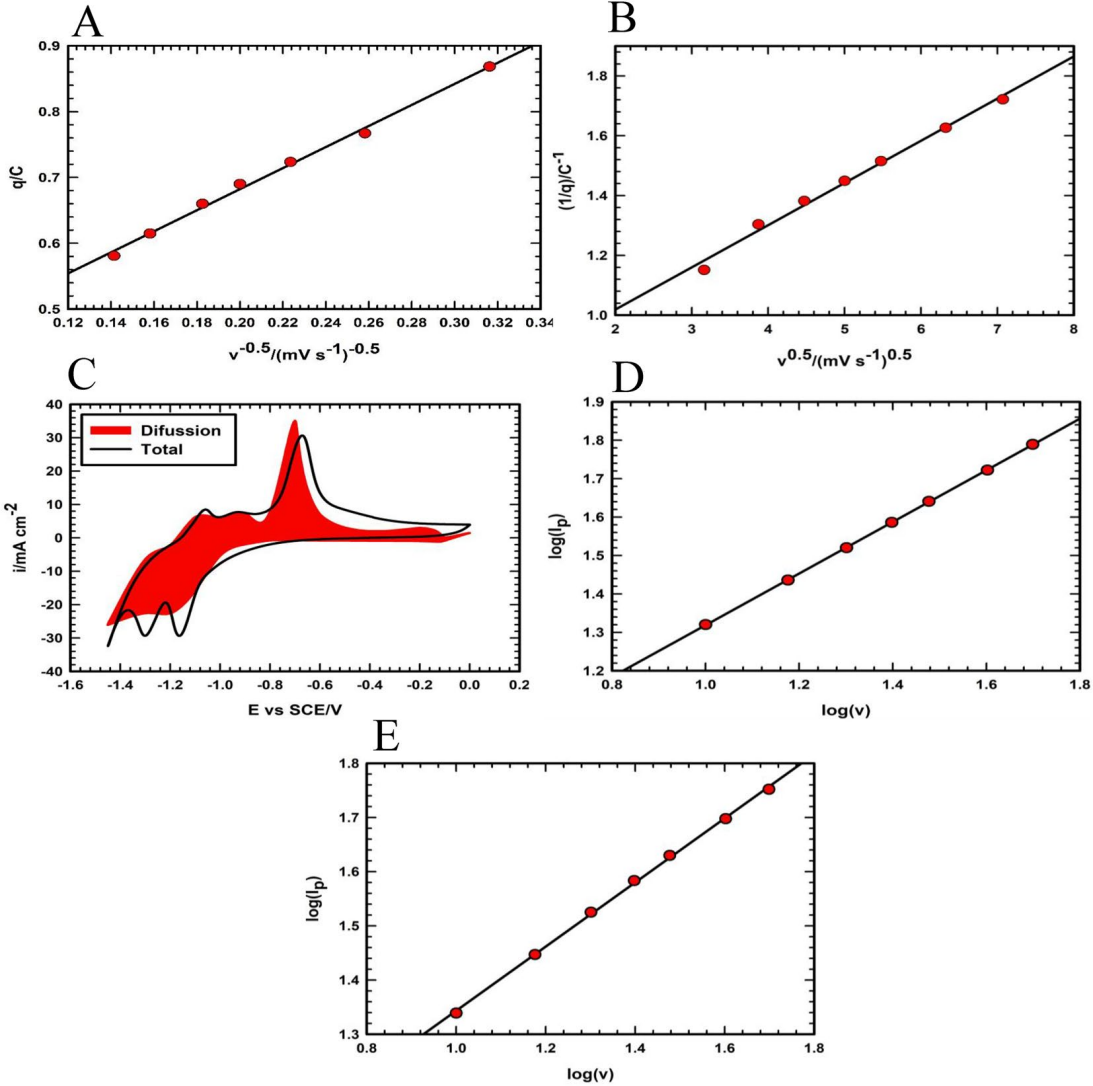
Trasatti method for the surface (**A**) and bulk reaction (**B**) of Fe_3_O_4_/GF electrode. Dunn method for determining bulk contribution at a potential scan rate of 10 mV s^−1^ (**C**). The variation of log(*Ip*) with log(v) of I (**D**) and V (**E**) peaks (from Fig. 7A) of the Fe_3_O_4_/GF electrode.

Interestingly, the origin of the storage mechanism of the synthesized electrode can be investigated by the analysis of the CVs at various potential sweep rates. Therefore, the Trasatti method is introduced to calculate the percentage of the contribution of the different storage processes of the synthesized materials using Eqs. 6 and 7^5,6,65^:

6 $$q={q}_{\infty }+a{\nu }^{-0.5}$$

7 $$\frac{1}{q}=\frac{1}{{q}_{t}}+b{\nu }^{0.5}$$

where *q* represents the charge (C) at various potential scan speeds (*ν*), a and b are constants, *q*_*∞*_ denotes the charge owing to the surface process (fast surface faradaic and adsorption of electrolyte ions), and *q*_*t*_ is the total charge (bulk and surface processes). The values of *q*_*∞*_ and *q*_*t*_ are 0.362 and 1.36 C, respectively, based on the intercept values of Fig. 9A,B. The ratio of the surface process (surface faradaic and ions adsorption) to the overall charge is 26.6% suggesting the combination storage mechanism during the charging and discharging of Fe_3_O_4_/GF electrode materials. But the Trasatti method gives this ratio based on the extrapolation of *q*_*∞*_ and *q*_*t*_ at the *ν* equal ∞ and 0, respectively. Thus, the storage mechanism of the active materials over the selected potentials sweep rates is mainly dependent on the intercalation and de-intercalation within the active materials and this is clear from the appearance of various redox peaks of CVs of Fe_3_O_4_/GF. Also, this behavior is confirmed by change separation using the Dunn method^66–68^. Figure 9C displays that the percentage of bulk faradic process is the main percentage of storage mechanism. Interestingly, at high-speed scan (50 mV s^−1^) the bulk faradic reaction contributes by 67.2%.

Furthermore, the bulk faradaic process may be further analyzed by using the relation concerning redox peak current and the potential scan rate using Eq. 8^69^:

8 $${I}_{p}=a{\nu }^{b}$$

where *I*_*p*_ is the redox peak current, *v* is the potential sweep rate, and a and b are constants. In this instance, the b-value represents the rate at which the electrolyte ions inside the active materials intercalate and de-intercalate throughout the charging and discharging process. For the diffusion-controlled process (battery behavior), the b-value is equal to 0.5, while for the intercalation pseudocapacitive, it is equal to 1. Bot mechanism, i.e. Bulk faradaic and intercalation pseudocapacitive, is suggested when the b value is between 0.5 and 1. The b-value, which is ascribed to the relation between the redox current of peaks I and V (from Fig. 7A) with the potential scan rate, was obtained from the slopes of Fig. 9D,E and are found to be 0.67 and 0.59, respectively. One can conclude from b values that the bulk faradaic process is mixed between intercalation and diffusion-limited mechanism. However, the predominant mechanism is the diffusion-controlled process due to the values being close to 0.5.

**Figure 10:**
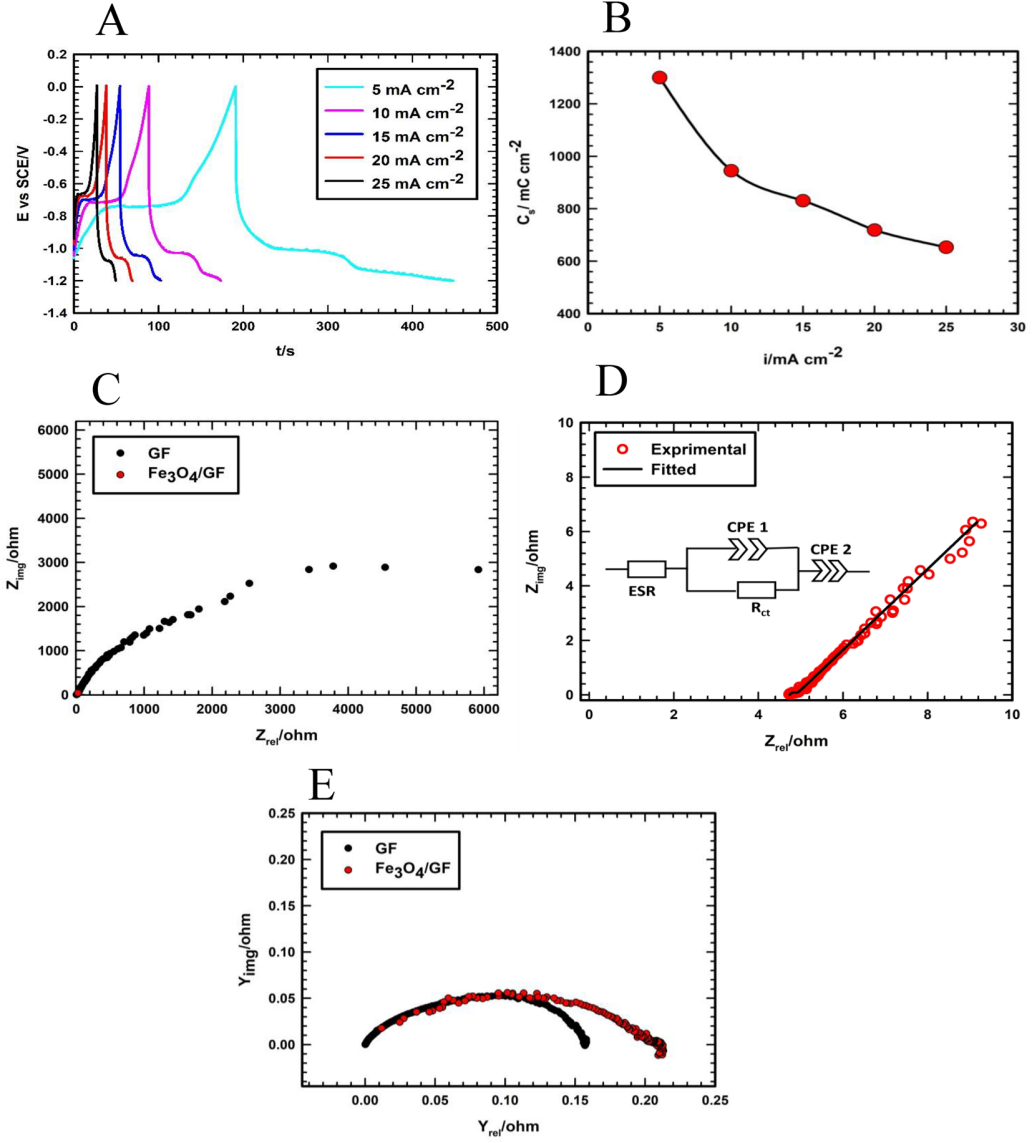
(**A**) GCD curves of Fe_3_O_4_/GF electrode at various current densities. (**B**) The variation of C_s_ of Fe_3_O_4_/GF electrode with various current densities. (**C**) Nyquist plots of GF and Fe_3_O_4_/GF electrodes at OCP (**D**) Nyquist for the Fe_3_O_4_/GF electrode. (**E**) Admittance plots of GF and Fe_3_O_4_/GF electrodes at OCP.

Secondly, the GCD test is obtained for Fe_3_O_4_/GF at various current densities. Figure 10A shows that the shape of the GCD curve is a battery-like electrode by displaying the plateau which is consistent with the data and results obtained from the investigation of CVs of the Fe_3_O_4_/GF electrode. Figure 10B displays the variation of *C*_*s*_ with various current densities and the obtained values are 1300, 944.7, 830.33, 718.4, and 653 mC cm^−2^ at current densities of 5, 10, 15, 20, and 25 mA cm^−2^, respectively.

To further emphasize the improving function of the preparation process and to provide clarification for the previously described findings, EIS measurements of the pristine support and Fe_3_O_4_/GF electrodes are conducted. From Fig. 10C, the Fe_3_O_4_/GF displays the steeper and shorter length line in low frequency region in comparison with pure GF demonstrating the excellent capacitive performance of the Fe_3_O_4_/GF electrode. Additionally, from Fig. 10D and its inset, the equivalent circuit of Fe_3_O_4_/GF electrode (inset of Fig. 10D displays four components: equivalent series resistance (ESR) (is represented by the intercept in the real axis at the high-frequency region), constant phase element concerning double layer capacitance (CPE 1), charge transfer resistance (*R*_*ct*_), and constant phase element concerning the capacity of (CPE 2))^70,71^. Interestingly, the encasement of Fe_3_O_4_ active materials around GF fibers makes the ESR of GF increase resulting in a decrease in the total electronic mobility. However, compared to the GF electrode, the Fe_3_O_4_ electrode has a lower ESR value. This suggests that the preparation method improves the GF material's electronic conductivity. The admittance plot (see Fig. 10E) provides additional confirmation of this.

As a consequence of the Fe_3_O_4_/GF electrode's electrochemical studies above, which are conducted as a negative electrode for SC applications. the Fe_3_O_4_/GF electrode compared to other related Fe_3_O_4_-based materials shows distinct features. The characteristics and features of the prepared electrode materials are concise as follows: (i) it is prepared with just one inexpensive, and simple electrochemical procedure, (ii) binder-free method, (iii) in contrast to the other work, where a pretreatment step is used, the GF is in-situ altered, improving both ionic and electronic conductivity, and (iv) Fe_3_O_4_/GF has an exceptionally broad potential window for operation (1.45 V). However, the prepared electrode suffers from limited stability due to deterioration of the swelling and shrinking of the electrode materials throughout the cyclic performance which may be improved in future work by addressing many parameters such as the incorporation of other metals to develop a binary system that can relieve the stress during the cyclic performance, and coating the active materials with the above layer from carbon material that is increasing the stability without affecting the other parameters.

## Conclusion

The easy synthesis of Fe_3_O_4_ nanostructure with distinctive storing performance is the focus of this work besides the investigation of its storage mechanism. The proposed method has many advantages over the other reported methods for the preparation of the Fe_3_O_4_ at the surface of the modified carbon support. Both the in-situ surface alteration of GF and Fe_3_O_4_ nanowires deposition occurred in a single simple procedure that was energy conservative, inexpensive, safe, and time-efficient. Various analyses were utilized to emphasize the deposition of the nanostructure of the prepared materials at the in-situ altered GF surface. Advantageously, inserting the functional groups at the GF surface provides a homogenous encasement of Fe_3_O_4_ around GF fibers and accelerates the contact of the active materials with electrolyte ions. Interestingly, the Fe_3_O_4_/GF electrode has a unique storage performance over a wide potential window up to 1.45 V, which is considered one of the widest potential windows for the Fe_3_O_4_ in the negative potential region, especially in the alkaline medium. Fe_3_O_4_/GF electrode displayed a specific capacity of 1418 mC cm^−2^ at a potential scan rate of 10 mV s^−1^ and this value retained to 54% at a potential scan rate of 50 mV s^−1^ over an extended potential window of 1.45 V. The higher storage performance is attributed to the high deposited material over the large GF area, high accessibility of ions, and enhanced electronic mobility. Furthermore, the mechanistic investigation reveals that the quick surface redox reaction and the diffusion-limited interaction between the electrolyte ions and the Fe_3_O_4_ redox active sites combine to form the produced electrode's storage mechanism. However, the predominated mechanism is the limited diffusion reaction. Thus, this electrode can be used efficiently with the positive electrode that is based on the capacitive operation to make an efficient asymmetric SC device with a wide potential window.

## Data Availability

The data that support the findings of this study are available from the corresponding author upon reasonable request.
